# Label Distribution in Tissues of Wheat Seedlings Cultivated with Tritium-Labeled Leonardite Humic Acid

**DOI:** 10.1038/srep28869

**Published:** 2016-06-28

**Authors:** Natalia A. Kulikova, Dmitry P. Abroskin, Gennady A. Badun, Maria G. Chernysheva, Viktor I. Korobkov, Anton S. Beer, Eugenia A. Tsvetkova, Svetlana V. Senik, Olga I. Klein, Irina V. Perminova

**Affiliations:** 1 Lomonosov Moscow State University, Department of Soil Science, Leninskie Gory 1-12, 119991, Moscow, Russia; 2 Bach Institute of Biochemistry, Research Center of Biotechnology of RAS, 33, bld. 2 Leninsky Ave., Moscow 119071, Russia; 3 Lomonosov Moscow State University, Department of Chemistry, Leninskie Gory 1-3, 119991, Moscow, Russia; 4 Lomonosov Moscow State University, Department of Biology, Leninskie Gory 1-12, 119991, Moscow, Russia; 5 Zelinskii Institute of Organic Chemistry of RAS, 119991, Moscow, Russia; 6 Komarov Botanical Institute of RAS, 2 Professor Popov str., St. Petersburg, 197376, Russia

## Abstract

Humic substances (HS) play important roles in the biotic-abiotic interactions of the root plant and soil contributing to plant adaptation to external environments. However, their mode of action on plants remains largely unknown. In this study the HS distribution in tissues of wheat seedlings was examined using tritium-labeled humic acid (HA) derived from leonardite (a variety of lignites) and microautoradiography (MAR). Preferential accumulation of labeled products from tritiated HA was found in the roots as compared to the shoots, and endodermis was shown to be the major control point for radial transport of label into vascular system of plant. Tritium was also found in the stele and xylem tissues indicating that labeled products from tritiated HA could be transported to shoot tissues via the transpiration stream. Treatment with HA lead to an increase in the content of polar lipids of photosynthetic membranes. The observed accumulation of labeled HA products in root endodermis and positive impact on lipid synthesis are consistent with prior reported observations on physiological effects of HS on plants such as enhanced growth and development of lateral roots and improvement/repairs of the photosynthetic status of plants under stress conditions.

Crop productivity is dependent on the capability of plants to adapt to external environments. Humic substances (HS) are the major components of soil organic matter^1^,^2^ which play important roles in the biotic-abiotic interactions of the root plant and soil contributing to plant adaptation^3^. Due to complexity of HS structure, exploration of their mode of action on plants has been a vivid field of the research for more than hundred years^3^,^4^,^5^,^6^,^7^,^8^,^9^,^10^,^11^. The most documented effect is stimulation of the root growth including root hair formation^10^ and lateral root development^11^ which was first reported in the pioneering work by Khristeva^5^.

Recently the extensive reviews on the effects of HS on plant metabolism were published^12^,^13^. A variety of enzymes were identified which are involved in a plant response to HS action including plasma H^+^–ATPase^14^,^15^,^16^, H^+^–pyrophosphatase^17^, Fe(III) chelate-reductase^14^, glycolytic enzymes, and enzymes of the tricarboxylic acid cycle^18^. A consensus was achieved with regard to multiple regulating functions of HS including direct stimulation of root growth and root hair proliferation, modulation of the release of protons and root exudates, regulation of ion-uptake rates, redox reactions, and others^13^. However, the further progress in this direction is limited by a lack of data on the primary targets of HS action.

To overcome this problem, a transcriptomic approach was applied, which does not require a preliminary hypothesis on the mode of action of HS^12^,^19^. It was demonstrated that HS affected expression of 133 genes in *Arabidopsis thaliana* responsible for the processes of binding, catalytic activity, and activity of transporters. The authors hypothesized that HS influence plant development by interfering with the transcription of genes involved in meristem formation and organization, cell cycle, microtubule organization and cytokinesis. Still, they could not surmise exact mechanisms of HS effects on plant physiology from the obtained results. This is also because the authors used whole homogenized plants for their studies when spatial variation in transcripts which could be seen in the different tissues and cell types is largely obscured or lost. In addition, the minor cell components inherent to only certain cell types are thereby strongly diluted, particularly when those cells represent a very small portion of the organ^20^. Therefore, genetic studies could add a value *via* the knowledge of spatial distribution of HS among the plant cells and tissues.

To facilitate this task, direct observations of HS entry into the root interior, as well as on transport and spatial distribution among the plant tissues are needed. The previous studies have confirmed feasibility of HS uptake by plants relying on a use of ^14^C-labelled synthetic humic materials^6^,^7^,^21^. These studies have shown that both low and high molecular weight fractions of HS were capable to enter the plant interior, but the larger penetration was observed for the lighter fraction. This was confirmed by the studies on HS conjugated with fluorescein isothiocianate (FITC), which was performed on cultured carrot cells^22^. Only the low molecular weight humic fraction was able to interact with the plasma membrane of cultured carrot cells. In our recent studies, we used tritium-labeled HS^23^ to demonstrate that the wheat seedlings accumulated labeled products in the roots, and they were able to translocate the minor portion into the shoots. Moreover, the analyses of lipid fraction extracted from the treated seedlings revealed that the tritium label was present mainly in the neutral lipid fraction consisting of alkanes and alkenes, which are usually found in plant waxes, associated with the cuticle and suberized tissues. However, direct influence of HS on the plant lipid biosynthesis still remains unclear.

Here, we will further exploit an advantage of using the labeled humic materials for elucidating the spatial distribution of the uptaken HS in the treated plants by a use of microautoradiography (MAR) and evaluate influence of HS on the content of plant polar and neutral lipids. Used in this study as a model HS is a humic acids (HA) fraction derived from leonardite (a variety of lignite), which is the major source for commercial humates applied in agriculture. In addition, physiological activity of leonardite HA is widely known^24^,^25^,^26^. The isolated HA is treated by using thermal bombardment to ensure incorporation of tritium label into the non-exchangeable sites of humic backbone as described previously^27^. This technique provides for even distribution of the label among all molecular weight fractions of the humic materials. To avoid overinterpretation of the obtained MAR images, we assign the observed patterns to distribution of either “the tritiated humic products” ([^3^H]HPs), or “tritium label”, rather than to distribution of the tritiated HA themselves. This is because we cannot exclude that the tritiated HA are transformed by plant or microbial metabolism during their uptake and utilization or that only some fractions of HA might be uptaken by the plant. The corresponding abbreviation [^3^H]HPs is used throughout the manuscript.

## Results

### Characterization of parent and tritium-labeled HA

**Figure 1:**
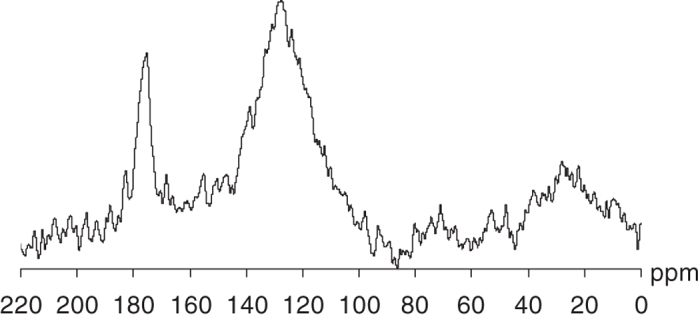
^13^C NMR spectrum of the leonardite HA used in this study.

The HA fraction extracted from leonardite was used for tritium-labeling. The HA isolate was characterized by prevailing contribution of aromatic structures as follows from the corresponding ^13^C NMR data (Fig. 1, Table 1). The ^13^C NMR spectrum was typical for leonardite HA whose most peculiar feature is domination of aromatics-related structural groups^28^. The content of aromatic carbon in the isolated HA accounted for 52.5%. This is consistent with the data of elemental analysis which yielded H/C value of 0.87 (Table 1) indicative of high unsaturation degree and hydrophobicity of the humic material used in this study.

**Figure 2:**
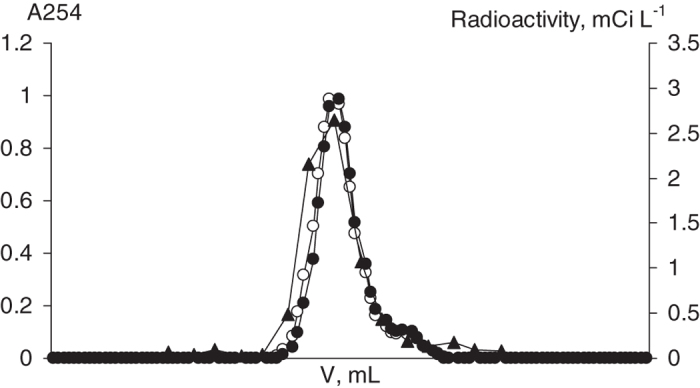
SEC profiles of the parent leonardite HA and [^3^H]HA obtained by dual detection of UV absorbance at 254 nm (left ordinate) and of radioactivity counting (right ordinate). The UV-profile of the parent HA is shown by open dots, [^3^H]HA - by black dots. Radioactivity profile of [^3^H]HA is shown by black triangles.

To monitor possible alteration during the labeling procedure, the comparative analysis of molecular weight distributions within the parent and labeled HA ([^3^H]HA) was performed using size-exclusion chromatography (SEC). To detect HS at the column exit we registered UV-absorbance and radioactivity of the eluate as described in our previous studies^27^. The data are presented in Fig. 2.

The UV-profiles for HA and [^3^H]HA were characterized with very similar monomodal distributions indicating a lack of significant change during labeling procedure. Of particular importance is that UV-profile of [^3^H]HA was also identical to the radioactivity profile indicating even distribution of tritium label among HA fractions with different molecular weights.

### [^3^H]HPs distribution in the tissues of the treated wheat plants as followed by film MAR

**Figure 3:**
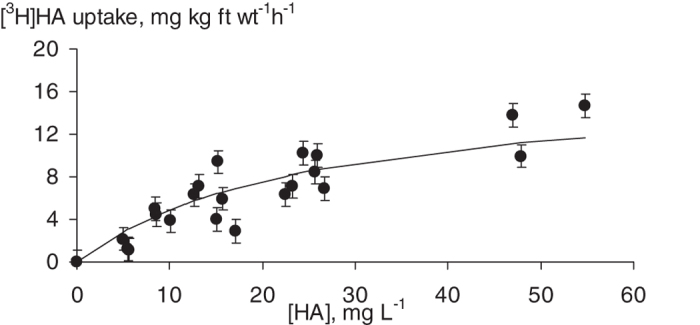
Concentration dependence of [^3^H]HPs uptake by wheat seedlings. Bars represent standard deviation, fr wt, fresh weight.

To select HA concentration for studies on tissue distribution of plants study HA entry and accumulation in plants, wheat seedlings were cultivated with different concentrations of [^3^H]HA varied from 5 to 60 mg L^−1^. The tested HA concentrations laid in the range of those occurring in soil solutions. They are more physiologically relevant as compared to the higher concentrations, which might cause phytotoxic effects to plants. Accumulation of [3H]HPs in wheat plants was characterized with smooth saturation curve reaching plateau at about 50 mg L^−1^ (Fig. 3). Based on these results, we have cultivated wheat plants for the tissue distribution experiments at this HA concentration.

**Figure 4:**
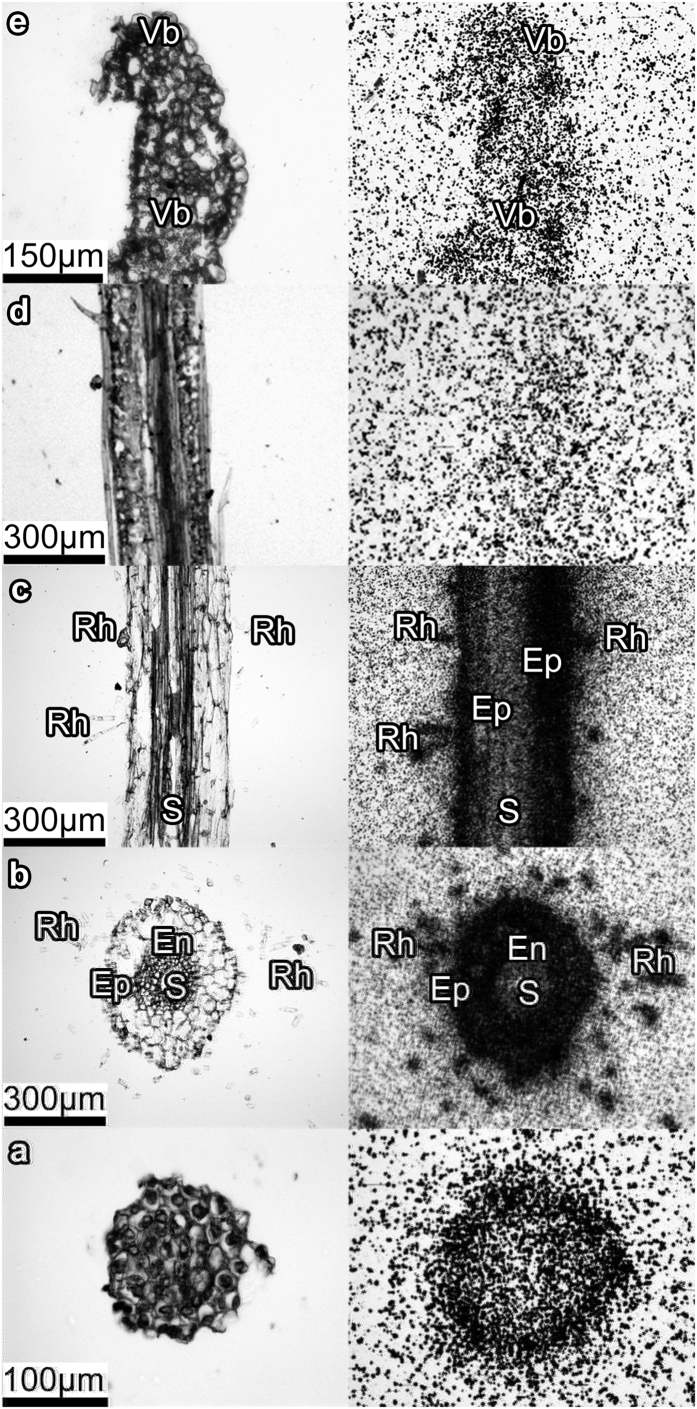
Light microscope images (left) and the corresponding tritium sensitive film MARs (right) of the slides with the cross-sections of the eight-day old wheat seedling treated with [^3^H]HA (50 mg·L^−1^, ~0.7 mCi L^−1^, 24 h). Exposure time for MARs was 97 days. (**a**) the transverse section of the shoot tip; (**b**) The longitudinal section of the leaf blade; (**c**) the longitudinal section of the root elongation zone; (**d**) the transverse section of the root elongation zone; (**e**) the transverse section of the root cap. Vb – vascular bundle; S – stele (**c,d**) Rh – root hair; Ep – epiblema; En – endodermis. The sections were alphabetically labelled with regards to the upward water movement in the plant from the root to the shoot.

The [^3^H]HA-treated plants were microtomed into different root and shoot zones (e.g., root cap, root hair zone, root elongation zone, middle part of leaf blade, and leap apex) and filmed using tritium sensitive film. At the identical exposure time of the film, blackening of the root sections (Fig. 4c–e) was much stronger as compared to the shoot ones (Fig. 4a,b). This could be indicative of preferable accumulation of the label by the root: only small portion of the label was available for the transfer into the shoot along the vascular system. The similar phenomenon was observed by Nardi *et al*.^3^ who reported that the amount of humic materials transferred from pea roots to the shoots did not exceed 10–12% wt.

Inspection of the different sections of the root has shown that for the zones with meristematic (undifferentiated) cells present (e.g. root cap, Fig. 4e) the label was adsorbed only at the outer layer. On contrary, a significant amount of the label was accumulated in the zones with differentiated cells (e.g., root hair zone, Fig. 4c,d). It can be seen that in the root hair zone the label entered the stele through the epiblema and cortex. Once inside the stele, the label moved upward into the xylem vessels, and then, to the shoots. Such a route is confirmed by the presence of the label in the xylem (Fig. 4c,d), and shoot apices (Fig. 4a). At the same time, the concentration of the label was extremely low in the plant shoot (Fig. 4b) indicating a crucial role of endodermis in impeding translocation of the [^3^H]HPs into the plant vessels.

Similar to the root zone, the distribution of [^3^H]HPs within the shoot (Fig. 4a,b) was not homogeneous with preferred accumulation in the leaf tip (Fig. 4a) and in the vascular system (Fig. 4c,d). The observed pattern could be related to retardation of the labeled products in the upper part of longitudinal veins due to increased concentrations of the label in the xylem sap associated with water evaporation. As a result, the label accumulation in the leaf tip could be explained by the high level of vascularisation in that region in monocots.

### [^3^H]HPs distribution in the plant tissues as followed by nuclear emulsion MAR

**Figure 5:**
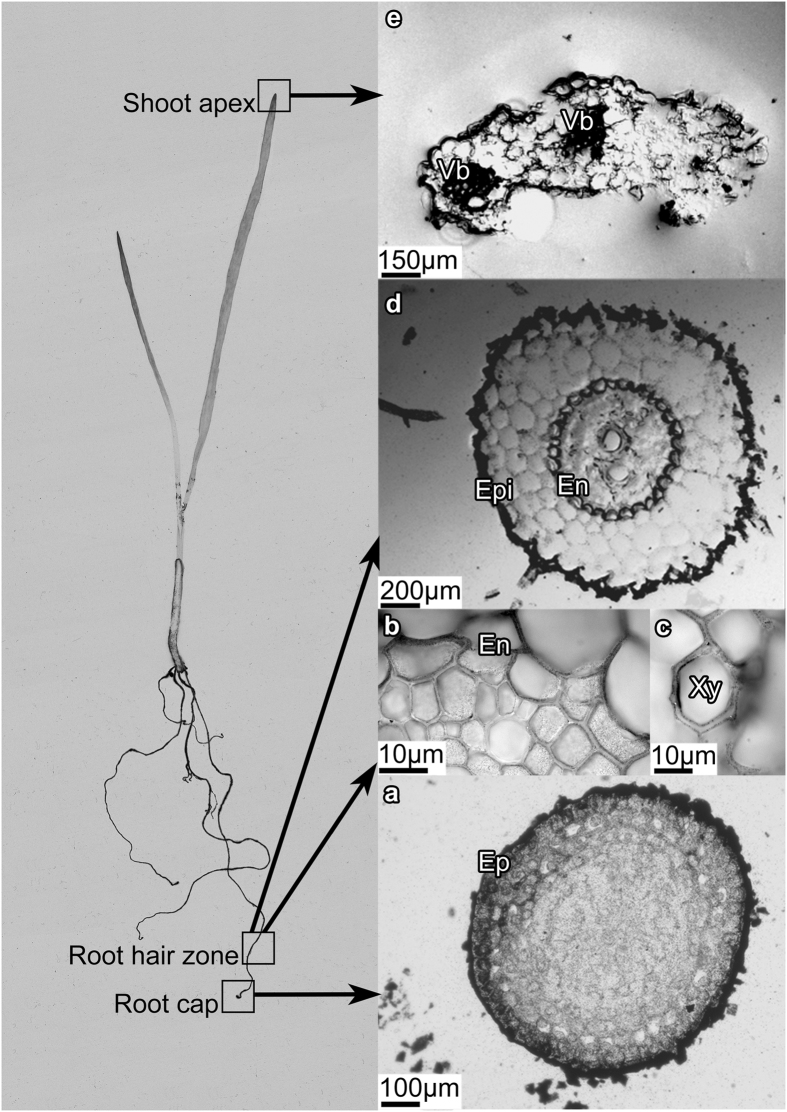
The autoradiogram of the whole wheat plant (left panel) and nuclear emulsion MARs of the slides with the cross-sections of the eight-day old wheat seedling treated with [^3^H]HA (50 mg·L^−1^, ~0.7 mCi L^−1^, 24 h) (right panel). Exposure time for the MAR was 97 d. (**a**) Transverse section of the shoot apex; (**b–d**) transverse section of the root hair zone; (**e**) transverse section of the root cap.

**Figure 6:**
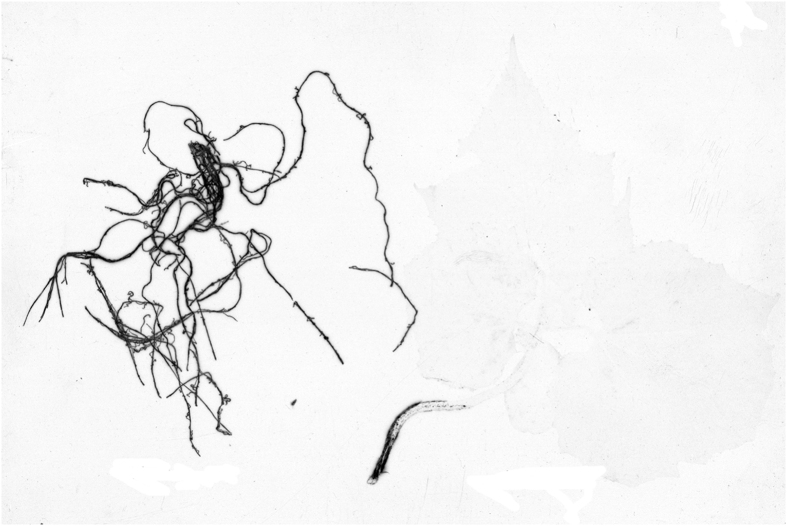
The autoradiograms of the whole cucumber plants treated with 50 mg L^−1^ [^3^H]HA. Exposure time was 5 d.

To overcome low resolution of tritium-sensitive film, which is not sufficient for detecting the label distribution within the plant tissues at the cellular level, the MAR experiments were performed using tritium sensitive nuclear emulsions. The slides with root sections were used for this purpose, whereas the sections of the medium part of leaf were excluded due to very low radioactivity. The corresponding emulsion MARs are shown in Fig. 5 (right panel) along with an autoradiogram of the whole wheat plant with the highlighted locations where root and leave cross-sections were sampled from (left panel). For comparison, autoradiogram of the whole cucumber plant is given in Fig. 6. Autoradiograms of both wheat and cucumber plants were characterized with similar features of the label distribution: it has mostly accumulated in the roots with much smaller portion translocated in the shoots. It was displayed as black-colored root zone and faintly colored leaves.

Deeper insight into the label penetration into the plant could be obtained from MARs of microtomed wheat plants shown in right panel of Fig. 5. Sharp contrast in the color of the outer and inner zones of the root cap can be observed in Fig. 5a and might be indicative of the preferred accumulation of [^3^H]HPs at the root surface, and of minor penetration of the tritium label into the root interior. This pattern corroborates well the film images of the same root section (Fig. 4e). The MAR of the hair root zone demonstrate darkening of the xylem vessels (Fig. 5c) which might be indicative of the label penetration into the vascular system. This is in line with the images obtained for the leaf tips (Fig. 5d), which displays characteristic darkening in the vessels. Besides, strong tritium signal was also detected in the leaf epidermis including trichome (Fig. 5d,e). Of particular importance is the substantial darkening in the region of the endodermis, which indicates strong accumulation of the label by endodermis (Fig. 5b) demonstrating its crucial role in the radial transport of [^3^H]HPs into the vascular system of the plant.

The nuclear emulsion MARs were characterized with the lower intensity of [^3^H]HPs in the cortex region, whereas both epiblema and endodermis were darkly colored (Fig. 5b). This is different from the film MARs which showed high intensity throughout the whole cortex region. The observed differences might be explained by lower sensitivity of the nuclear emulsion as compared to the film. Therefore, [^3^H]HPs were seemingly accumulated mainly by epiblema and endodermis rather than uniformly distributed in the root. It is of interest given that the root endodermis is characterized by the presence of Casparian strip and suberin lamellaes which serve as two hydrophobic barriers restricting the free diffusion of molecules between the inner cell layers of the root and the outer environment. Another hydrophobic region where strong signals of [^3^H]HPs were observed was leaf epidermis (Fig. 5d). This might be indicative of preferred accumulation of [^3^H]HPs onto lipophilic cell wall barriers including both suberin-(endodermis) and cutin (exoderma). To explore on that, we have undertaken further examination of HA influence on lipid profile.

### Influence of the leonardite HA on lipid profile of wheat seedlings

**Figure 7:**
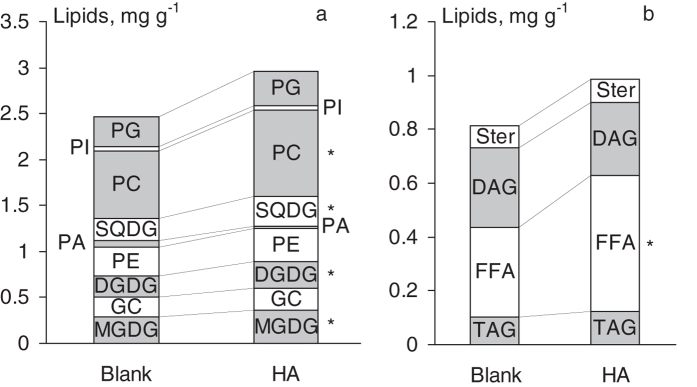
Influence of the leonardite HA on profiles of polar (**a**) and neutral (**b**) lipids in the treated wheat seedlings. *Different from blank at α = 0.05.

The following polar lipids were found in the wheat plants used is this study: monogalactosyldiacylglycerol (MGDG), digalactosyldiacylglycerol (DGDG), glucosylceramide (GC), phosphatidylcholine (PC), phosphatidylethanolamine (PE), phosphatidic acid (PA), phosphatidylinositol (PI), phosphatidylglycerol (PG), and sulfoquinovosyl diacylglycerol (SQDG). The plants treatment with the leonardite HA caused statistically significant increase in MGDG, DGDG, SQDG, and PC (Fig. 7a).

Neutral lipids were presented by triacylglycerol (TAG), diacylglycerol (DAG), sterols (Ster) and free fatty acids (FFA). Introduction of the leonardite HA did not alter the content of TAG, DAG, and Ster, but it induced an increase in the content of FFA (Fig. 7b).

## Discussion

The results of the undertaken autoradiographic examinations of the wheat plants treated with the labeled leonardite HA have demonstrated substantial variations in penetration pattern of the [^3^H]HPs into the different plant zones. Autoradiogram of the whole wheat plant (Fig. 5) showed much higher accumulation of the label in the roots as compared to the shoots. Of importance is that similar pattern was observed in the whole cucumber plant when used in the same experiments: much stronger darkening of the roots as compared to the shoots (Fig. 6). This might be indicative of the common features in HS penetration into monocotyledonous plants (wheat) and dicotyledonous plants (cucumber). Deeper insight into the tissue distribution of the labeled humic materials was obtained upon examining microtomed sections of the wheat plants. In the root cap zone, the label did not penetrate into the plant interior and was mostly absorbed by the root epiblema (Figs 4e and 5a). In the root hair zone, which is the central entry pathway for nutrients into the plant, the strong tritium signals were observed both in the epiblema and endoderma (Figs 4d and 5b). In the stele, the presence of solid black spots indicated the radial transport of [^3^H]HPs (e.g., HA, some of their fractions, or metabolized products) into the wheat plant vasculature. The observed accumulation of the label on epiblema and endoderma as well as a lack of the label in the root tip interior conforms to predominantly apoplastic pathway of the radial transport of [^3^H]HPs. For a univocal conclusion on the entrance of the humic materials into the vascular system of the plants, a special study is needed.

The results obtained allow us to assume that both the epiblema and the endodermis act as filters for the [^3^H]HPs resulting in accumulation of [^3^H]HPs in these zones and entrance of only particular fractions of the humic materials into the vascular system of the plants. According to the modern view in the field, the endodermal membrane together with the Casparian strips form a tight barrier, which regulates the apoplastic pathway, thus forcing the solutes to move through the selectively permeable plasma membrane into the cytoplasm^29^. The important consequence is that radial transport of molecules with high molecular weights (higher than that of a tracer dye propidium iodine, 668 Da) is restricted at the outer side of the endodermis, and they can not diffuse radially in the cell wall space across the endodermis^30^. Only relatively small molecules could be therefore expected to be found in the stele region of the root. The same might be true for humic materials: fractionation of their molecular constituents might occur along the passage from outer solution into the xylem. This suggestion is in line with our previous data on the presence of [^3^H]HPs in the neutral lipid fraction of the plants treated with the HA which consisted mostly of alkanes and alkenes^23^. This finding corroborates well the data of the current study on the elevated concentrations of tritium label in the cuticle and suberized tissues (Fig. 5) which might be indicative of the important role of HA in the lipid metabolism in plants.

The direct evidence of this statement is a significant increase in the content of some polar (MGDG, DGDG, SQDG, and PC) and neutral (FFA) lipids detected in the presence of HA (Fig. 7). Two of them (galactolipids MGDG and DGDG) are known as the predominant components of the photosynthetic membranes, particularly in thylakoids, where they compose 50 and 20% of the polar lipids, respectively. MGDG is also found in the reaction centers of the photosystem I and II, which is indicative of its role both as a bulk constituent of thylakoid membrane and as an integral component of the photosystem complexes^31^. The third lipid (SQDG) can be found exclusively in the chloroplast membrane: both in the thylakoid membrane (but at lesser quantities compared to the galactolipids) and in the chloroplast envelope; it is crucial for functional and structural integrity of the photosystem II complex^32^. The phospholipid PC plays a key role both in membrane structuring and glycerolipid biosynthesis being a principle site of fatty acid desaturation and a precursor of chloroplastic lipids in leaves^33^. The enhanced synthesis of glycolipids and PC in the wheat seedlings in the presence of HA might be indicative of stimulating action of humic material on plant photosynthesis. The latter is in good agreement with the numerous findings on intensified photosynthetic processes under the influence of HS^3^. At that, low concentration of [^3^H]HPs in the green parts of the plant points to the fact that HA induced biosynthesis of the above mentioned polar lipids. Unlike polar lipids, FFA are major components of suberin and cutin waxes^34^. Therefore, an increase in FFA in the presence of HA along with the observed elevated concentration of [^3^H]HPs in the cuticle and suberized tissues might be indicative of a possible role of HA in suberin and cutin biosynthesis.

Collectively, results of preferable accumulation of the labelled products of leonardite HA in the root endodermis and increase in polar lipids of the photosynthetic membranes in the presence of leonardite HA are consistent with prior reported observations on HA physiological effects on plants such as enhanced growth and development of lateral roots and improvement/repairs of the photosynthetic status of plants under stress conditions. Given that any other than humic acid fraction (e.g. fulvic acid, hymatomelanic acid) derived from any other than leonardite source (e.g. peat, sapropel, soil) can be labeled with tritium, the prospects of using labeled humic materials for investigating the mode of their action on plants appear promising. In the future, of particular interest might be investigations of [^3^H]HPs distributions in plant tissues targeted for transcriptomic studies. The gained knowledge could be implemented for developing advanced rhizosphere management practices aimed at improving plant growth via educated use of humics-based green agrochemicals.

## Methods

### Humic acid isolation and characterization

Leonardite HA was isolated from potassium humate produced by Humintech Ltd. (Germany). A weight of potassium humate was dissolved in distilled water and centrifuged (30 min, 3000 *g*) to separate insoluble components. The supernatant was acidified to pH 2.0 with concentrated HCl and centrifuged (30 min, 3000 *g*). The HA precipitate was collected, washed with distilled water, desalted by dialysis (2 kDa cut-off membrane, Merck, Germany) against distilled water, evaporated at 60 °C, and stored in dissicator over P_2_O_5_.

Structural characterization of the isolated HA included elemental analysis, potentiometric titration, size-exclusion chromatography (SEC) and ^13^C NMR spectroscopy (Table 1). Elemental analysis (C, H, and N) was conducted using Vario El Microcube analyzer. Ash content was determined by manual combustion at 850 °C. Oxygen content was calculated as a difference between a mass of the sample and the found amount of ash and CHN. The contents of all elements were calculated on ash-free basis. The content of acidic groups was determined using potentiometric titrations as described by Balcke *et al*.^35^.

The SEC analysis was performed as described by Perminova *et al*.^36^. Toyopearl HW-50S gel was used for column packing. Sodium salts of polystyrenesulfonic acid of molecular mass of 2.29, 4.48, 14.0, 20.7, 45.1, and 80.8 kDa (Polymer Standard Service, Germany) were used as markers for molecular mass calculations. HA solution was diluted to a concentration of 40 mg·L^−1^ with the SEC mobile phase (0.028 M phosphate buffer, pH 6.8) before analysis. Based on the data obtained, weight-averaged molecular weight (M_W_) was calculated using GelTreat software^37^.

Quantitative ^13^C solution-state NMR spectra were recorded on an Avance NMR spectrometer (Bruker, Germany) operating at 100 MHz carbon-13 frequency. A 50 mg HA sample was dissolved in 0.6 mL 0.3 N NaOD and transferred into a 5 mm NMR tube. ^13^C NMR spectra were acquired with a 5-mm broadband probe, using CPMG pulse sequence with nuclear Overhauser effect suppression by the INVGATE procedure; the acquisition time and relaxation delay were 0.2 s and 7.8 s, respectively. These conditions allowed quantitative determination of carbon distribution among the main structural fragments of HA^38^. The assignments were as follows (in ppm): 5–108, aliphatic non-substituted, O- and N-substituted C atoms (∑C_Alk_); 108–165, aromatic non-substituted, O- and N-substituted C-atoms (∑C_Ar_); 165–187, C atoms of carboxylic and ester groups (C_COO_); 187–220, C atoms of quinonic and keto- groups (C_C=O_).

### Preparation and characterization of tritium-labeled humic acids

Tritium-labeled humic materials ([^3^H]HA) were prepared as described by Badun *et al*.^27^. Briefly, 1 mL of 0.3 g L^−1^ HA solution in 0.005 M NaOH was uniformly distributed on the wall of the reaction vessel, and then frozen with liquid nitrogen and lyophilized. The reaction vessel was placed under vacuum, filled with tritium gas (0.5 Pa) and the tungsten filament in the central part of reactor vessel was heated to 1950 K. The HA was treated with tritium atoms for 10 s. The residual gas was evacuated and a new portion of tritium gas was introduced for further labeling if necessary. The obtained [^3^H]HA samples were dissolved in 0.005 M NaOH and purified by dialysis (2 kDa cut-off membrane, Merck, Germany) against phosphate buffer (0.028 M, pH 6.8) at 4 °C for 1 month. This procedure eliminated exchangeable tritium from the OH, COOH, and NH_n_ groups of HA. The radioactivity of solutions of labeled substances was measured using a liquid scintillation spectrometer (RackBeta 1215, Finland).

To monitor possible alteration of HA due to partial decomposition or polymerization during the labeling procedure as well as to monitor tritium-tracer distribution among HA fractions of different molecular weights, comparative analysis of parent HA and [^3^H]HA was performed using SEC analysis according to the procedure described above. Both, UV and radioactivity detection were applied. To register radioactivity profiles of [^3^H]HA, 2 mL fractions were collected during the SEC experiment and analyzed for radioactivity. UV-detected chromatograms of both parent HA and [^3^H]HA exhibited single coincident peaks. Thus, one can conclude that no significant changes in HA molecules occurred during the reaction with atomic tritium. On the other hand, UV-profile of [^3^H]HA coincided with radioactivity profile. The latter was evident for regular distribution of tritium among HA fractions differing in molecular weight^27^.

### Plant cultivation

Seedlings of wheat *Triticum aestivum* L. (cv. Inna) were used for the experiments. Wheat seeds were germinated in the dark at 24 °C during 72 h. Then, the germinated seedlings were transferred into 0.5 L polyethylene tanks containing Knop nutrition solution (KH_2_PO_4_ 0.14 g·L^−1^, KCl 0.1 g·L^−1^, KNO_3_ 0.14 g·L^−1^, MgSO_4_ × 7H_2_O 1.42 g·L^−1^, Ca(NO_3_)_2_ × 12H_2_O 4.88 g·L^−1^, FeCl_3_ × 6H_2_O 0.05 g·L^−1^, pH 5.5) and placed into the growth chamber (12/12 hr photoperiod, illumination 200 μmol m^−2^ s^−1^; 24 °C) for 72 h.

For uptake experiments, six-days old plants were transferred into the HA-containing vials with specific radioactivity of ~0.07 mCi L^−1^ (vials volume was 15 mL, HA concentrations varied from 5 to 60 mg·L^−1^). Five plants were used per one vial and exposed for 24 h. Then, the plants were taken out of the HA-containing solutions, whose residues were drained from the roots. To estimate label uptake by plants, radioactivity was measured before and after plant growth within 24 h using liquid scintillation method. The data were plotted into sorption isotherms. All experiments were performed in five replicates.

For autoradiography measurements, the six days old plants were cultivated as described above at one concentration of HA of 50 mg·L^−1^.

### Microautoradiography protocol

For the MAR studies, eight-days old plants were transferred into vials (three plants per each vial) which were filled with 15 mL of HA at a concentration of 50 mg·L^−1^ with specific radioactivity of ~0.7 mCi L^−1^. Twenty four hours later the plants were sectioned using standard paraffin embedding and serial sectioning at 15 μm thickness as described by Ruzin^39^. Then sections were put on microscope slides (Roth, Karlsruhe, Germany) and treated with chloroform to remove paraffin. Obtained slides were then subjected to MAR analysis using the tritium-sensitive X-ray film Kodak Biomax (Kodak, USA) or dipped in undiluted NBT-3 film emulsion (Kodak, Rochester, NY) and stored at 4 °C in the dark for 105 days before developing. Time of exposition was determined in preliminary experiments (data not shown). Film or emulsion development, fixing, and washing were performed according to procedure recommended by the manufacturer. Slides with plant sections were imaged with a Zeiss Axioplan 2 imaging microscope (Zeiss, Germany) equipped with a Zeiss AxioCam MRc color video camera, and Zeiss Axiovision 3.1 software.

### Lipid extraction and quantitative lipid analysis

After harvesting the shoots of plants, lipid extracts were obtained with hot isopropanol according to the Nichols method^40^. Concentrated lipid preparations were fractionated with thin-layer chromatography (TLC) on silicagel 60 10 × 10 cm plates (Merck, Germany). Polar lipids were fractionated by two-dimensional TLC in a solvent system chloroform–methanol–water (65:25:4, v/v) in the first direction and chloroform–acetone–methanol–acetic acid–water (50:20:10:10:5, v/v) in the second direction^41^. Neutral lipids were fractionated by one-dimensional high performance TLC (HPTLC) with double development. Toluene–hexane–formic acid (140:60:1, v/v) and hexane–diethyl ether–formic acid (60:40:1, v/v) mixtures were used as the mobile phases^42^. Phosphatidylcholine, monogalactosyldiacylglycerol, ergosterol, and triacylglycerol (Sigma, UK) were used as markers used for TLC. The amounts of glycero- and sphingolipids were determined densitometrically using a Denscan device (Lenchrom, Russia) after visualization by heating with a 5% H_2_SO_4_ solution in methanol. The Mann-Whitney U test was used to compare amounts of lipids in the blank and HA-treated plants using on-line Mann-Whitney U Test Calculator at http://www.socscistatistics.com/tests/mannwhitney/Default2.aspx. Significance level α was 0.05.

## Additional Information

**How to cite this article**: Kulikova, N. A. *et al*. Label Distribution in Tissues of Wheat Seedlings Cultivated with Tritium-Labeled Leonardite Humic Acid. *Sci. Rep.* **6**, 28869; doi: 10.1038/srep28869 (2016).

## Figures and Tables

**Table 1:** Structural characteristics of the leonardite HA used in this study.

Elemental composition			M_W_, kD	Acidic groups, mg-eqv g^−1^		Carbon content in the structural fragments, %			
H/C	O/C	C/N		COOH	PhOH	C_C=O_	C_COO_	∑C_Ar_	∑C_Alk_
0.87	0.50	53	9.9	4.2	1.1	4.4	14.1	52.5	28.9
